# Birds Shed RNA-Viruses According to the Pareto Principle

**DOI:** 10.1371/journal.pone.0072611

**Published:** 2013-08-21

**Authors:** Mark D. Jankowski, Christopher J. Williams, Jeanne M. Fair, Jennifer C. Owen

**Affiliations:** 1 United States Fish and Wildlife Service, Pocatello, Idaho, United States of America; 2 Department of Zoology, University of Wisconsin-Madison, Madison, Wisconsin, United States of America; 3 Department of Statistics, University of Idaho, Moscow, Idaho, United States of America; 4 Biosecurity and Public Health, Los Alamos National Laboratory, Los Alamos, New Mexico, United States of America; 5 Department of Fisheries and Wildlife, Michigan State University, East Lansing, Michigan, United States of America; 6 Department of Large Animal Clinical Sciences, Michigan State University, East Lansing, Michigan, United States of America; Northeast Agricultural University, China

## Abstract

A major challenge in disease ecology is to understand the role of individual variation of infection load on disease transmission dynamics and how this influences the evolution of resistance or tolerance mechanisms. Such information will improve our capacity to understand, predict, and mitigate pathogen-associated disease in all organisms. In many host-pathogen systems, particularly macroparasites and sexually transmitted diseases, it has been found that approximately 20% of the population is responsible for approximately 80% of the transmission events. Although host contact rates can account for some of this pattern, pathogen transmission dynamics also depend upon host infectiousness, an area that has received relatively little attention. Therefore, we conducted a meta-analysis of pathogen shedding rates of 24 host (avian) – pathogen (RNA-virus) studies, including 17 bird species and five important zoonotic viruses. We determined that viral count data followed the Weibull distribution, the mean Gini coefficient (an index of inequality) was 0.687 (0.036 SEM), and that 22.0% (0.90 SEM) of the birds shed 80% of the virus across all studies, suggesting an adherence of viral shedding counts to the Pareto Principle. The relative position of a bird in a distribution of viral counts was affected by factors extrinsic to the host, such as exposure to corticosterone and to a lesser extent reduced food availability, but not to intrinsic host factors including age, sex, and migratory status. These data provide a quantitative view of heterogeneous virus shedding in birds that may be used to better parameterize epidemiological models and understand transmission dynamics.

## Introduction

In the last century, there has been an unprecedented increase in the numbers of emerging infectious diseases (EIDs), which pose significant risks to wild and domestic animal and human populations [1]. The goal and biggest challenge to health professionals is to predict and slow the course of a disease epidemic and minimize the number of affected individuals. Predicting the spread of a disease and changes in the number of infected individuals within a population is typically performed using epidemiological models, [2], [3] which track the number of susceptible, infected, and recovered individuals. However, these models frequently assume a homogeneous population in which the ‘infected’ are equally infectious. Yet, we know that populations are heterogeneous and that individuals vary in their ability to maintain pathogens, with some individuals exhibiting high pathogen loads (i.e. ‘supershedders’, [4]) while others maintain average or low pathogen loads.

The importance of transmission heterogeneity to the spread of disease is becoming increasingly recognized [5]–[7]. Nevertheless, our understanding is limited because it is not well studied [8]. The best illustration of the importance of heterogeneity in host response to pathogens is the incidence of superspreaders [2], [9], in which 20% of a host population contributes to 80% of transmission. Pathogen superspreading can be linked to disproportionate contact rates, heterogeneous pathogen load, or an interaction between these factors. However, to date, evidence for superspreaders has been primarily associated with an increase in contact rates and behavior of the individuals [10], rather than variation in a host’s infection intensity. Yet, given the same exposure rate we know that individuals vary in the ultimate pathogen load that they develop [7], [11], [12].

Quantitative information concerning the heterogeneity of viral load across infected individuals in a population would be the first step to establishing a link between supershedders and superspreaders. In this paper, we comprehensively evaluated the inequality of viral load and present a meta-analysis of 24 avian-virus experimental infection studies, which included 17 species of birds and five RNA viruses of three different families, Orthomyxoviridae, low and high pathogenic avian influenza virus (LP- and HPAIV); Flaviviridae, West Nile virus (WNV) and St. Louis encephalitis virus (SLEV); and Togaviridae, eastern equine encephalitis virus (EEEV) and western equine encephalitis virus (WEEV). RNA viruses in particular, are thought to be the causative agent for approximately 30% of the identified EIDs [13], [14].

The role of birds in the ecology of a variety of diseases of public health concern is well documented [15], [16]. Birds account for 10.3% of zoonotic and 18.4% of emerging zoonotic diseases [17] and serve as the natural reservoir for several pathogens of economic and public health importance, including the above agents and Japanese encephalitis virus [9], [13], [18]. Birds can also contribute to the geographic spread of pathogens through migration [16], [19], [20]. Thus, an understanding of bird-virus interactions not only informs basic questions concerning host-pathogen biology, but will also aid in understanding the risk of disease to humans.

The specific goals for this paper were to: (I) test the hypothesis that viral load in birds is consistently heterogeneous by (a) determining which data distribution function best fit viral load data (e.g., Weibull or Pareto) and (b) measuring the degree of inequality (via Gini coefficients) of viral load across individuals within an experimental study population; (II) evaluate factors associated with high viral shedding (supershedding); and, (III) indicate how our observations can be used to further understand and control avian-borne viral zoonotic diseases. We have found heterogeneous viral shedding patterns to be consistent across all 24 datasets evaluated. Based on our findings, we identify future research directions to understand the mechanisms driving these patterns as well as potential ways to link these findings with disease prevention and control strategies.

## Materials and Methods

### Experimental Viral Infection Studies

We identified experimental infection studies from a variety of host-pathogen (bird-virus) systems that satisfied the following criteria: (a) individuals in each experiment were infected with a known dose of a viral pathogen (i.e., exposure was controlled), (b) the experimental birds were wild-type or wild-caught, so as to represent natural genetic variation, (c) pathogen load in the avian host was monitored for the entire infectious period, and (d) host pathogen load was measured at an anatomic site most relevant to transmission for a given host-pathogen system. The data represent a variety of published studies (mean n = 20.7 SEM 3.3) that were conducted by different laboratories and individuals (see cited papers for experimental methodology; but, see Nemeth et al. [21] for WNV inoculation and viral plaque methods used in the budgerigar (*Melopsittacus undulates*) study). We use the term ‘shedding’ to reflect the transmissible fraction of virus burden, whether it is circulating virus in the blood (viremia) or actively deposited virus into the environment.

### Data Analysis

To quantify an individual’s relative pathogen load, we analyzed the data in the following manner: for a given experiment, we performed calculations of individual and group pathogen load and plotted as an empirical CDF according to Lorenz [22]. We define individual pathogen load as the total number of pathogen particles (e.g., one plaque forming unit (PFU)/ml for live virus or one copy of viral RNA) detected in an experimental subject throughout the duration of the infection period, while the group’s pathogen load is simply a summation of individual pathogen loads. In a Lorenz curve, the x-axis is the percent cumulative birds and the y-axis is the percent cumulative group virus.

We first identified studies (four) with greater than 30 subjects to evaluate the distribution of the data as described below. The remaining 20 datasets with fewer than 30 subjects were not used for distribution fitting. These data were then analyzed using R (R Foundation for Statistical Computing, 2.15.1) for their probability distribution and degree of inequality as follows.

1. Determination of distribution functions and parameter estimates: The distribution functions for each of the four studies having greater than 30 subjects were determined by maximum likelihood estimation methods. Candidate probability distributions were Pareto, Weibull, Generalized Extreme Value (GEV), and exponential (EXP). These distributions each model long-tailed data via differing distribution functions and parameters. Using the ‘fitdistr’ function library ‘MASS’ in R [23], the scale, shape, or location parameters were estimated as appropriate given the distribution being tested (Weibull, GEV or EXP). The shape parameter estimate (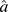) for the Pareto distribution was calculated manually as 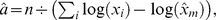 in which *n* is the sample size, x_i_ is a vector of data, and 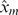 is the minimum value for a given empirical distribution (i.e., the location parameter). Using the generated parameter estimates each dataset was visually checked for fit against each statistical distribution by quantile plots and this was followed by the calculation of the Anderson-Darling goodness of fit statistic, which is biased towards the tails of data and thus appropriate for the current analysis. The ‘ad.test’ function in package ‘ADGofTest’ in R [24] was used for this task. After the best statistical distribution was determined with the four studies (n>30), parameter estimates (shape and scale) were generated for all remaining 20 datasets (n<30).
2. Inequality of infection: We quantified the inequality of infection via the Gini coefficient using the ‘gini’ function in the ‘reldist’ package in R [25]. The Gini coefficient is commonly used to quantify inequality of income across different human populations but has also been used to quantity the differential reproductive success of parasites in wildlife [26], [27]. It is a standardized index, which compares the area under the curve (AUC) of Lorenz (cumulative distribution) curves [22] drawn under the assumption of perfect equality of a resource across individuals or groups to the AUC of the curve for the distribution under investigation. It is calculated as, Gini = 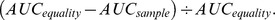 Therefore, for a population under study, a value of 0.0 indicates perfect equality and a value of 1.0 indicates a maximum concentration of a substance.
3. Lastly, to facilitate an intuitive understanding of these results, we determined 50^th^, 80^th^ and 90^th^ virus shedding percentiles for each host-pathogen system.

We next evaluated factors associated with the inequality of infection (i.e., Gini coefficient). The effect of anatomic site in which virus was measured, host species, virus species, or the number of individuals in the experimental infection study were evaluated for their effect on Gini (Wilcoxon or Kruskal-Wallis for one or multiple levels of a factor, respectively) in SAS JMP 8.0 (Cary, NC). Finally, we binned birds according to their relative virus shedding quantity (< or ≥80^th^ percentile, the latter were then classed as “supershedders”, see Discussion) and then tested for the effect of intrinsic (sex, age, migratory status) and extrinsic (virus dose, exogenous corticosterone treatment, food availability) factors on their status as a supershedder (yes or no) by logistic regression using SAS JMP 8.0. Odds ratios were estimated when a factor was determined to be statistically significant (*P*<0.05) for its effect on a bird’s classification as a supershedder.

## Results

**Figure 1:**
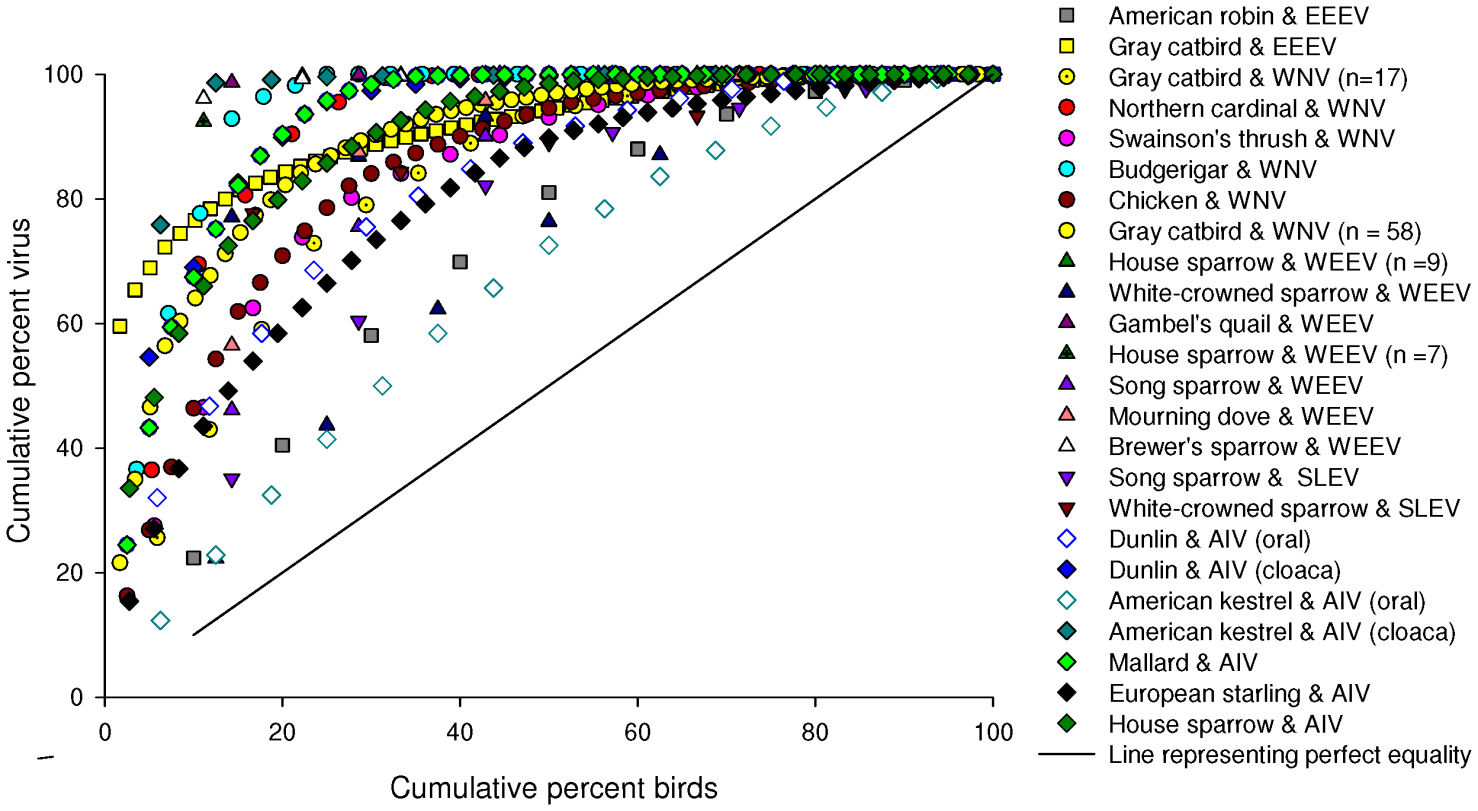
Cumulative distribution functions (i.e., Lorenz curves) for 17 different bird species infected with different viral pathogens (as noted), demonstrating that most of the virus shed by an infected population was detected in a minority of the individuals in that population. The x-axis is percent of total potentially transmissible group virus and the y-axis is the percent of the total number of birds. Symbol shape is grouped by pathogen and symbol color is grouped by species. For AIV in dunlin and American kestrel we summed the samples for oral and cloacal swabs separately. Replicate species-virus curves represent different experimental infection studies that differ by date, location of the study and sample size (as indicated in parentheses).

Across all 24 datasets and 20 unique avian-virus systems, we found that avian viral loads were not equally distributed across individuals and best fit a long-tailed data distribution function (Figure 1). The distribution of the data of the four avian viral infection datasets tested (i.e., those studies with *n* ≥30) best fit the Weibull distribution (Anderson–Darling goodness of fit test, *P*>0.05). These data followed no other distribution that we evaluated (i.e., Pareto, exponential, or generalized extreme value). We subsequently estimated the scale (i.e., the spread of the data) and shape (i.e., slope of the line) parameters for these and each of the 20 remaining datasets (i.e., those studies with *n* <30) (Table 1). The estimated shape parameter 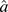 was less than 1.0 (mean 0.66 SEM 0.08), indicating that most of the virus was shed by a few individuals. The parameter estimate 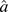 was not sensitive to the mean viral count or sample size (linear regression with log-normal errors, *P*>0.05).

**Table 1:** Quantitative description of unequal virus shedding across 24 avian-virus infection datasets.

Avian host*	Virus†	Site	*n*	κ‡	κ (SEM)	λ§	λ (SEM)	Gini¶	50^th = |^	80^th^	90^th^	Reference
American kestrel	HPAIV	cloaca	16	0.48	0.13	10937170.0	16777.2	0.903	50.0	12.5	12.5	[35]
Brewer’s sparrow	WEEV	Serum	9	0.22	0.05	433.9	681.6	0.878	44.4	22.2	11.1	[59]
House sparrow	WEEV	Serum	9	0.22	0.05	352500.0	NA	0.871	44.4	22.2	11.1	[59]
Budgerigar	WNV	serum	28	0.32	0.04	20.9	13.3	0.867	25.0	18.0	7.0	(Bowen R & NemethN, personal comm.)
Gambel's quail	WEEV	serum	7	0.23	0.06	583.7	969.6	0.853	42.9	28.6	14.3	[59]
Dunlin	HPAIV	cloaca	20	0.38	0.06	61.7	38.4	0.836	45.0	20.0	10.0	[34]
Mallard	LPAIV	cloaca+oral	40	0.26	0.03	52790.9	NA	0.831	50.0	20.0	10.0	[31]
Northern Cardinal	WNV	serum	19	1.07	0.13	0.9	0.1	0.812	47.4	21.1	10.5	[60]
Gray catbird	EEEV	serum	58	0.57	0.05	3217000.0	5932.0	0.808	50.0	20.7	10.3	[61]
House sparrow	LPAIV	oral	36	0.47	0.06	94.1	35.3	0.775	50.0	19.4	11.1	[62]
Gray catbird	WNV	serum	59	0.42	0.06	258981.0	34006.0	0.758	50.0	20.7	10.3	[29]
House sparrow	WEEV	serum	7	0.48	0.13	10936000.0	16780.0	0.732	42.9	28.6	14.3	[59]
Mourning dove	WEEV	serum	7	0.43	0.13	442112.7	16778.3	0.684	42.9	28.6	14.3	[59]
Chicken	WNV	serum	40	0.62	0.08	13566.0	3555.0	0.673	50.0	20.0	10.0	[42]
Swainson's thrush	WNV	serum	18	0.64	0.11	965300.0	8389.0	0.648	50.0	22.2	11.1	[19]
White-crowned sparrow	SLEV	serum	6	0.73	0.00	521064.3	NA	0.640	50.0	16.7	16.7	[59]
Gray catbird	WNV	serum	17	0.59	0.00	547670.0	NA	0.628	47.1	23.5	11.8	[19]
Dunlin	HPAIV	oral	17	0.76	0.14	7565.1	2525.0	0.604	47.1	23.5	11.8	[34]
Song sparrow	WEEV	serum	7	0.55	0.00	106950.3	NA	0.601	42.9	28.6	14.3	[59]
European starling	LPAIV	oral	36	0.88	0.11	1082.20	214.4	0.573	50.0	19.4	11.1	[62]
Song sparrow	SLEV	serum	7	1.13	0.33	1955000.0	NA	0.459	42.9	28.6	14.3	[59]
American robin	EEEV	serum	10	1.34	.34	255511.8	NA	0.400	50	20	80	[63]
White-crowned sparrow	WEEV	serum	8	1.00	0.33	5548000.0	NA	0.347	50.0	25.0	12.5	[59]
American kestrel	HPAIV	oral	16	2.02	0.41	60709.1	6959.1	0.298	50.0	18.8	12.5	[35]
Mean	HPAIV	oral	20.7	0.66	0.08	1506141.2	5348.8	0.687	46.4	22.0	11.8.	
SEM			3.27					0.036	1.1	0.9	0.4	

* Avian host species in the order of first presented in table, *Falco sparvius, Melospiza melodia, Passer domesticus, Melopsittacus undulatus, Callipepla gambelii, Calidris alpinam, Anas platyrhynchos, Cardinalis cardinalis, Dumetella carolinensis, Zenaida macroura, Gallus gallus, Catharus ustulatus, Zonotrichia leucophrys, Sturnus vulgaris, Turdus migratorius.*

† Virus abbreviations, AIV, avian influenza virus; SLEV, St. Louis encephalitis virus; WEEV, western equine encephalitis virus; WNV, West Nile virus.

‡ Weibull shape parameter.

§ Weibull scale parameter.

¶ Gini coefficient (0.0 indicates perfect equality and 1.0 is complete inequality).

^= |^Percent of birds within a group which shed virus at the 50^th^, 80^th^, or 90^th^ percentile.

Given that parameter estimation can be unstable for small sample sizes, we further quantified the inequality of viral load by constructing Lorenz curves and calculating Gini coefficients for each of the 24 datasets. The mean Gini coefficient was 0.687±0.036 SEM indicating a high degree of inequality of viral load and an adherence to the Pareto Principle in which ∼20% of the individuals (i.e., birds) in a population account (i.e., shed) for ∼80% of a material (i.e., virus) [9], [28]. Providing further support for the adherence to the Pareto principle, we calculated the 50^th^, 80^th^ and 90^th^ percentiles and found that a mean of 22.0% ±0.90 SEM of the birds accounted for 80% of the virus in an experimentally infected population (Table 1).

No factor tested statistically significantly affected Gini (Table 2). However, when AIV was sampled in the oral cavity, the Gini coefficient was 0.307 lower than when compared to the cloacal viral counts, irrespective of host species, but this was not statistically significant (χ^2^ = 3.43, df = 1, *P* = 0.064; Figure 1). The mean Gini for all studies of oral virus was 0.563 SEM 0.099 and 0.870 SEM 0.030 for cloacal virus. The other factors tested (sample size, host species, virus, treatment with extrinsic factor) did not statistically significantly affect Gini (Table 2). For instance, although the number of individuals in each study (sample size) varied widely across experiments (n = 6 to 59) it did not influence Gini coefficients, including two studies conducted by the same investigator that differed only in their sample size (West Nile virus-infected gray catbirds (*Dumetella carolinensis*), n = 17 and 59) [19], [29].

**Table 2:** Quantitative assessment of potential host and virus factors on the value of the Gini Coefficient for 23 avian-virus laboratory studies.

Factor	DF	χ^2^	*P* Value
Sample size	13	11.68	0.554
Host species	12	8.10	0.777
Virus	4	0.65	0.957
Anatomic location of shedding	1	3.43	0.064
Extrinsic factor*	1	0.11	0.743

* Extrinsic factors were treatment with corticosterone or food restriction.

**Figure 2:**
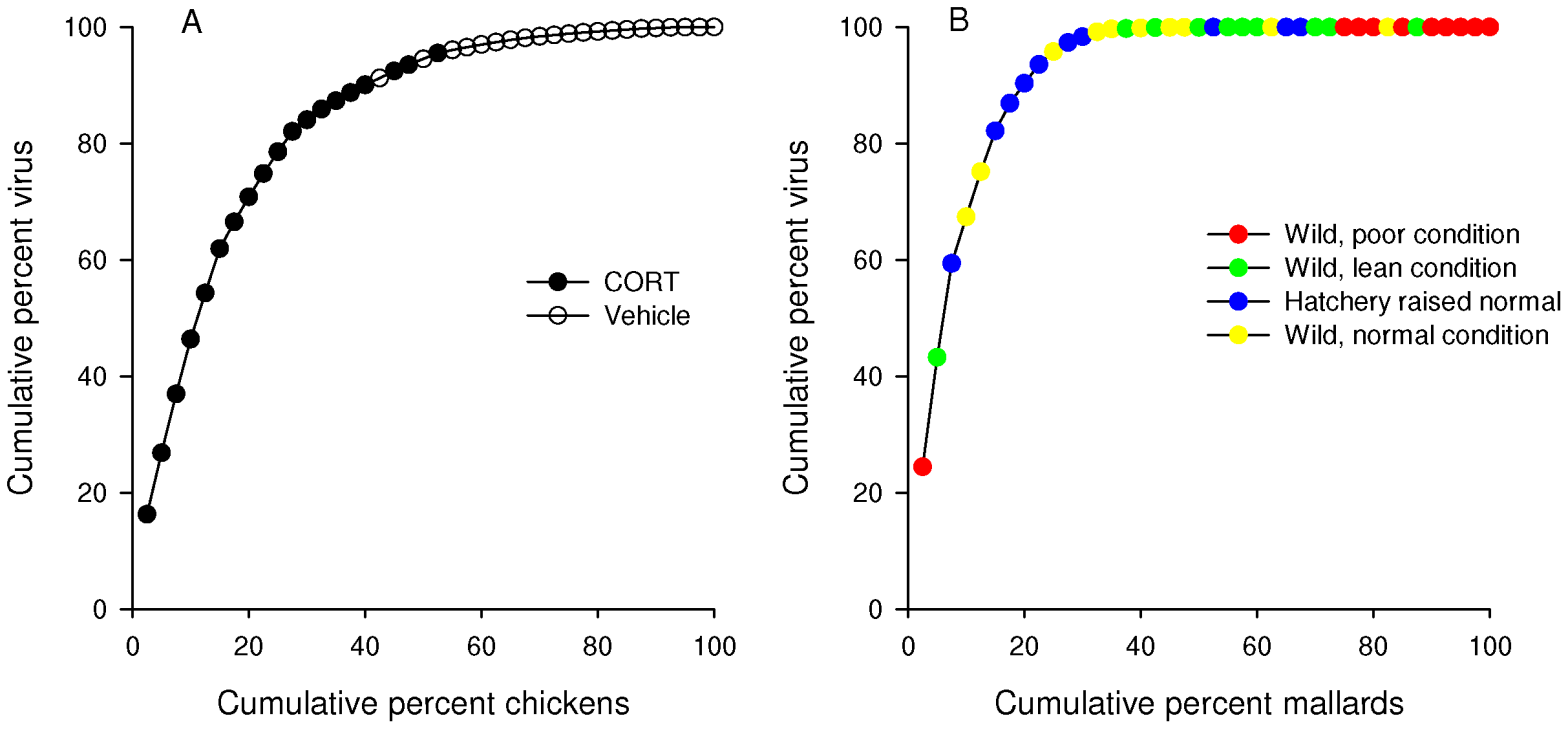
Factors (corticosterone and food availability) extrinsic to the host were associated with the position of a bird in the viral count distribution curves (i.e., Lorenz curve). (A) Impact of corticosterone (CORT) exposure compared to vehicle- (i.e., controls for a 0.1% ethanol solvent vehicle) treated birds on a West Nile virus-infected chicken’s (*Gallus gallus*) position in the Lorenz curve [42]. (B) Effect of varying food availability on an avian influenza virus-infected mallard’s (*Anas platyrhynchos*) position in the Lorenz curve [31].

Extrinsic but no measured intrinsic host factor affected the position of a bird in the distribution of viral counts (Figure 1; Table 3). Birds were categorized as supershedders if they shed at or higher than the 80^th^ percentile for a given study, placing them towards the left in the distribution of viral counts. No intrinsic factor evaluated (age, sex, migratory status) affected whether a bird was classed as a supershedder or not (Table 3). Of the extrinsic factors evaluated (corticosterone treatment, virus dose, and food availability), only corticosterone exposure positively impacted whether a bird was classed as a “supershedder” or not (χ^2^ = 17.33, df = 3, *P* = 0.0006). Specifically, the exposure of WNV-infected chickens to corticosterone statistically significantly affected whether a bird was a supershedder (i.e., shed at or higher than the 80^th^ percentile; χ^2^ = 14.17, df = 1, *P* = 0.0002; Figure 2A). A chicken exposed to corticosterone was more likely to be classed as a supershedder than a control chicken (Odds Ratio 320610; 95% confidence interval not estimable). Lastly, chickens exposed to corticosterone accounted for 93.4% of the total group virus but only 47.5% of the birds [30]. Interestingly, although the chickens in this study were domestically reared outbred birds, the level of shedding heterogeneity (Gini = 0.673) was within the range of wild-caught species infected with WNV (Gini of four WNV studies = 0.628–0.867). In another study in which food restriction treatments were used to achieve three body condition classes, body condition only moderately affected whether an AIV infected mallard (*Anas platyrhynchos*) was classed as a supershedder (χ^2^ = 3.13, df = 1, *P* = 0.077). A normally fed bird was 4.38 times more likely to be a supershedder than a bird maintained on a food-restricted diet (Odds Ratio 4.38; 95% CI, 0.85–33.25). However, while overall the birds in a state of reduced body condition contributed the least to the total group virus, the two highest shedders were in the lean and poor condition groups (Figure 2B) [31].

**Table 3:** The impact of extrinsic or intrinsic host factors associated with supershedding in avian-virus systems for which data were available (test statistics are shown for the whole model, and odds ratios with 95% confidence intervals, (CI) are shown only for selected factors (statistically significant factors are shown in bold text)).

Avian – Virus System	Extrinsic Factor	Intrinsic Factor	Interaction	n	χ^2^	*P* Value	Odds Ratio (95% CI)
Chicken – WNV	**CORT**	Sex	CORT×Sex	37	17.33	**0.001**	320610 (NE‡)
Kestrel – AIV (oral)	Virus Dose	Sex	Dose×Sex	16	2.02	0.568	
Kestrel – AIV (cloacal)	Virus Dose	Sex	Dose×Sex	16	3.34	0.342	
Dunlin – AIV (cloacal)	Virus Dose	–	–	20	0.115	0.735	
Dunlin – AIV (oral)	Virus Dose	–	–	17	0.267	0.606	
Catbird – EEEV	–	Sex	–	59	0.002	0.963	
Catbird – WNV	–	Sex	–	59	0.429	0.513	
Catbird – WNV	–	Migratory	–	17	0.018	0.893	
Cardinal – WNV	CORT	Sex	CORT×Sex	19	0.046	0.997	
Mallard – AIV	Food*	Sex	Food×Sex	40	4.87	0.181	4.38 (0.85–33.25)
Starling – AIV	–	Sex	–	36	1.789	0.181	
HOSP† – AIV	–	Age, Sex	–	36	0.702	0.704	

Notes:

* , Normally fed versus food-restricted mallards (10 & 20% body condition reduction, [31], χ^2^ = 3.12, df = 1, *P* = 0.077.

† , House sparrow.

‡ , Not estimable because the denominator was zero (i.e., no control treated birds were supershedders).

## Discussion

We present a synthesis of viral load in birds using 24 datasets and 20 unique avian-virus systems, which supports the conclusion that viral load follows the “20/80 rule” (i.e., the “Pareto Principle”) and that viral ‘supershedders’ consistently emerge in an infected population, regardless of avian host or viral species. Quantitatively characterized heterogeneity in virus load provides enormous utility for developing epidemiological models that are a better representation of reality [5], potentially leading to more effective and targeted disease control and treatment strategies through the identification of potential superspreaders [7], [9]. While the existence of supershedders has been demonstrated in other host-pathogen systems [4], [7], [11], [28], [32], there have been no reports of comprehensive studies on multiple host-pathogen systems as in the current paper.

Several lines of evidence in our study indicate that RNA-virus shedding in birds follows the Pareto Principle. First, it is visually evident that approximately 80% of the virus was shed by approximately 20% of the birds (Figure 1) and the data in Table 1 support this with an average of 22.0% of the birds shedding in the 80^th^ percentile. Second, the Gini coefficient was greater than 0.600, indicative of a high degree of inequality or the 20/80 Rule (Pareto principle). Third, the estimated Weibull shape parameter was less than 1.0, indicating a long-tailed data distribution. Hence, regardless of how we analyze the data we find consistent support for a skewed distribution of the data (i.e., the Weibull distribution) that can be described as following the Pareto Principle.

The good fit with the Weibull distribution likely reflects the time-dependent nature of viral burden and how it is quantified. The Weibull distribution models time-to-event data and virus is enumerated in a sample by counting plaques *in vitro* or as a copy of viral RNA via RT-PCR and the plaque count or copy number are then tracked during the infection period. The Weibull distribution is described by scale (λ) and shape (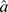) parameter estimates; the former is an indication of the mean viral count for the distribution and the latter describes the slope of the CDF plot. In general, scale is not easily interpretable across studies, and specifically this is because virus was measured from a variety of anatomic compartments and enumerated by many different assay techniques as described in the respective papers cited herein. However, the shape parameter estimate (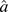) is more robust to comparisons across studies and datasets.

The mean shape parameter estimate (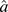) below 1.0 for the studies currently evaluated indicates that the majority of the virus was concentrated in a few individuals. Further, 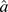 was not sensitive to the mean viral count or sample size. This lack of sensitivity to the mean is in contrast to previous descriptions of macroparasite load heterogeneity across wild organisms in which it was found that the negative binomial parameter (*k*) was sensitive to mean parasite burden [33]. However, given that parameter estimation is an imprecise exercise requiring very large sample sizes, significant problems have been identified with this way of measuring aggregation across study populations [27]. Moreover, because nuances of statistical distributions are not readily interpretable to all practitioners we additionally quantified the inequality of shedding via the Gini coefficient, providing us with an index to probe factors potentially influencing infection inequality across many studies.

We found little evidence of intrinsic or extrinsic factors influencing Gini (Table 2); mean viral burden (λ), sample size, host and virus species, host sex, anatomical site of collection (oral or cloacal for AIV), and two extrinsic factors (food availability and exogenous corticosterone) did not statistically significantly impact the value of Gini (Table 2). The Gini coefficient was higher, but not statistically significantly, for cloacal virus than it was for oral HPAIV H5N1 (A/whooperswan/Mongolia/244/05) in American kestrel (*Falco sparverius*) and dunlin (*Calidrisalpina appina*) [34], [35]. Therefore, we found the inequality of viral shedding to be a robust phenomenon.

The consistent inequality of viral shedding (i.e., Gini >0.600) across identically exposed hosts is a key finding because it suggests that host physiological and/or virus replication mechanisms rather than differences in exposure are associated with the emergence of differentially infected individuals. For host-pathogen systems in which supershedding individuals may disproportionately contribute to epidemics, this information would be of great epidemiological advantage, especially when these individuals can be identified before or early in the course of their infection and prior to transmission. Problematically, such early detection of highly infectious hosts is currently not possible because the mechanisms driving supershedding are largely unknown and grossly understudied, especially in epidemiologically relevant wild hosts.

### Defining “supershedder”

The transmission of an agent depends upon both contact rates between infected individuals or environments and uninfected individuals, and the ‘infectiousness’ of the infected individual. The latter depends on the amount of infectious pathogen an individual is circulating or sheds into the environment. Hence, superspreading is likely to depend on an individual’s propensity to ‘supershed’ infectious pathogen. Yet, quantitative definitions of both superspreading and supershedding have not been universally adopted. One definition that has been promulgated in recent studies [36] is that superspreaders are the individuals within a particular host-agent system that account for the *n*th percentile (e.g., 99^th^ percentile) of the transmission events [7]. Lloyd-Smith et al.’s [19] approach depends upon reliable estimates of the probability distribution (e.g., Poisson) of the basic reproduction number (R_0_) for a given host-pathogen system; however, this is not known for most wild organisms. In contrast to superspreading, there is no accepted definition of supershedding, which may be the result of inadequate knowledge about the role of host infectiousness on agent transmission and R_0_ in the wild [7]. However, in one study, Matthews et al. [12] did find strong evidence for the importance of supershedding on transmission of *E. coli* in cattle. Specifically, they found that by identifying the 5% of the population with the highest mean infectiousness and targeting them for culling affected estimates of R_0,_ more than any other factor evaluated.

For the purposes of our study, we defined supershedding as those individuals shedding at the 80^th^ percentile. This definition takes into account the observed 20/80-distribution of virus shedding in the systems analyzed in this study, as well as practicalities of characterizing epidemiological parameters and host attributes potentially associated with these parameters in wild host-pathogen systems. If the goal is for enhanced understanding and mitigation of disease in animals, as well as humans, then determining the 20% of hosts responsible for 80% of the virus would be both highly advantageous as well as more cost-effective than the identification of the 1% of birds shedding the highest levels of virus [36].

### Identifying features of supershedding

The preemptive identification of viral supershedders and potential superspreaders is key for the early and rapid mitigation of a disease outbreak. Identifying host features associated with supershedding may facilitate more targeted research or disease control efforts, and this can only be accomplished with a thorough understanding of the mechanisms responsible for infection heterogeneity. The life cycle of a virus provides the basis for investigations of supershedding and includes (I) the initial exposure of a host, (II) entry into sites of virus replication within a host through barriers and receptors, (III) virus replication, and (IV) exposure of another host to the virus [37]. Differences between individuals in the transition-rate from one step of the cycle to the next likely accounts for infection heterogeneity. In the majority of the 24 datasets that we selected for our analysis, stages ‘I’ and ‘IV’ were held constant, indicating that differential entry or replication of a given virus accounted for the observed heterogeneity. In addition, we found that ‘virus dose’ did not impact Gini coefficients (Table 2) or the tendency to become a supershedder (Table 3). Therefore, we suggest that host or virus factors associated with steps ‘II’ and ‘III’ should be studied for their relationship to heterogeneous viral shedding and subsequent disease transmission.

Extrinsic Factors: In two of the studies evaluated, we examined the impact of environmentally relevant factors on shedding inequality. Animal populations encounter conditions that can enhance the secretion of glucocorticoid hormones (e.g., corticosterone in birds), [38], [39] and if elevated levels are sustained, anti-viral immunity can be suppressed [40]. Additionally, food availability in the wild is not spatially or temporally homogenous and this can lead to under- or malnourishment with subsequent reductions in body condition and potentially immunocompetence [41]. In one study included in our analysis to address this hypothesis, corticosterone was exogenously elevated in chickens prior to and during WNV infection. Treatment with corticosterone was found to “shift” the position of a treated bird to the left (Figure 2A) such that only treated birds shed at the 80^th^ percentile whereas no control birds shed at this level [42]. This finding indicates that factors inducing short-term (10 days) rises in corticosterone (such as social stressors or migratory stress) in WNV infected birds may cause supershedders to emerge. However, we emphasize caution in extrapolating this finding to wild disease systems given the potentially lower genetic variability of the domestic chickens used in the aforementioned study compared to wild avian hosts.

The second extrinsic factor that affected the position of a bird (mallard) in the CDF plot in our study was food availability, but in a manner contrary to our *a priori* predictions. That is, mallards that experienced reduced food availability and body condition, shed lower levels of LPAIV than birds provided with ‘normal’ food allowances [31] and were thus plotted to the right in the distribution (Figure 2B). However, it is notable that the two highest shedders were in the ‘lean’ and ‘poor’ condition groups. This finding suggests that factors other than food availability and body condition influence pathogen load, such as intrinsic factors.

Intrinsic Factors: The host’s response to parasite infection can also depend on intrinsic factors including genetics [43], [44], age [45], [46], social status [47], or annual cycle attributes such as reproduction [48], [49] and migratory status [50], [51]. Although these studies suggest a role for intrinsic factors on total viral load quantity, in our study bird age, sex, and migratory status were not associated with viral shedding at the 80^th^ percentile (“supershedders”) (Table 3). A lack of an association between supershedding and the intrinsic factors evaluated may be a result of the inadequacy of any one trait to capture the highly complex nature of an organism’s response to a virus. One implication of the current finding is that much work remains to identify intrinsic host factors associated with high and low viral load.

### Future Directions

Virulence, an emergent property, is a consequence of coevolutionary processes between hosts and parasites in the context of constantly changing environments [52]. Taking inspiration from plant biologists studying host-parasite interactions, vertebrate biologists have begun investigating virulence (a form of parasite fitness) and host fitness in the context of resistance and tolerance mechanisms. Resistance refers to mechanisms leading to reduced parasite fitness whereas tolerance mechanisms are associated with enhanced host fitness. For example, a resistant host would experience a lower pathogen load compared to higher loads in a non-resistant host. A tolerant host would experience a pathogen load that is not negatively correlated with host health [53]. With regards to the current study in which a subset of birds (∼20%) accounted for a majority (∼80%) of the virus, genetically based differences in resistance and tolerance mechanisms may have influenced this skewed distribution. The consistent existence of supershedders in our study may suggest divergent populations that differed by resistance and tolerance mechanisms. Our analytical framework provides a means to investigate these issues in an epidemiological context as we separated populations by their quantity of potentially transmissible virus. In addition to host-specific resistance or tolerance mechanisms, our study also suggests questions concerning how the evolution of virus populations may vary by host.

In the current study, viral strain and often exposure concentration were held constant within an experiment, meaning that viral evolution would likely be dependent more upon the selective pressures imposed by the host environment than an initial variation of viral traits [54], [55]. Studies examining the interplay between host defenses and the evolution of viral traits are therefore warranted. For example, given that corticosterone (and to some extent, food availability) affected the tendency of a bird to become a supershedder in the current study, such extrinsic factors and their effect on host physiology and thus selective pressures on the virus may lead to differences in viral evolution between similarly infected hosts [56], [57].

An additional point of interest is with respect to how virus supershedding events may differ in domestic compared to wild animal systems. Although the 20/80 pattern appears to be robust across the systems evaluated currently, the emergence and control of supershedding individual domestic birds may be more predictable and feasible, respectively, than in wild systems. The often-limited genetic variability of domestic birds may be associated with a more uniform shedding/infection response to a given extrinsic factor than in wild birds, but this certainly depends upon genetic variability at relevant loci of a given domestic bird population [58]. For example, although only corticosterone exposed domestic chickens shed WNV at the 20^th^ percentile, hatchery raised mallards infected with AIV and provided with a “normal” diet did not uniformly shed at a different rate than wild-collected mallards exposed to the same experimental conditions (Figure 2). We further note that control measures focused on supershedding individuals would be initially more tenable in domestic than in wild disease systems. In addition to the potential for a more predictable emergence of supershedders as noted above, such individuals could be more cost-effectively sequestered from the system than individuals in wild systems. However, in discovering that viral shedding occurs according to the Pareto Principle, we have identified an additional point of knowledge that may facilitate the development of control measures in wild systems. Further, in zoonotic disease systems uniquely hazardous to humans or for those viruses affecting species of conservation concern, the cost:benefit ratio of enacting control measures may shrink compared to less consequential viruses. Although we have identified what appears to be a generalized shedding pattern in RNA-virus infected birds, other systems (e.g., supershedding cattle infected with E. coli) [4] emphasize that a specific understanding is critical before efficacious control measures can be developed.

Given that we have specifically demonstrated that virus shedding is not equally distributed across equally (experimentally) infected birds and that we know that superspreading events can depend upon either or both contact rates and host infectiousness, we suggest the following research directions. (1) Understand the role of supershedding individuals on the potentiation of superspreading events for a given disease system including comparisons between vector-borne and directly transmitted viruses. (2) For a given disease system, determine whether heterogeneous contact rates and heterogeneous shedding independently, additively, or synergistically affect epidemiological rates such as R_0_. (3) In cases when differential pathogen shedding influences epidemic growth rates, examine the role of factors extrinsic and intrinsic to the host on the development of differential pathogen load, with the goal of early detection of supershedders. (4) Examine whether genetic or physiologic biomarkers exist for supershedders of a pathogen, which could be used for early detection and identification of highly infectious individuals. (5) Collect pathogen load data in diverse systems including agricultural animals, wildlife, and humans to investigate environmental correlates with the presence of supershedding individuals. The coupling of experimental investigations of the determinants of supershedding and transmissions dynamics with epidemiological modeling, could lead to the identification of a critical missing component in the mitigation of disease in both animal and human populations.
